# Importance of the reflective logbook in improving the residents’ perception of reflective learning in the dermatology residency program in Saudi Arabia: findings from a cross-sectional study

**DOI:** 10.1186/s12909-022-03948-w

**Published:** 2022-12-13

**Authors:** Hend M. Alotaibi, Ruaa Alharithy, Hala M. Alotaibi

**Affiliations:** 1 Dermatology Consultant and Assistant, Professor College of Medicine, King Saud University, Riyadh, Saudi Arabia; 2 Dermatology Consultant and Assistant Professor, Princess Nourah Bint Abdul Rahman University, Security Forces Hospital, Riyadh, Saudi Arabia; 3 Dermatology Resident, Saudi Board Dermatology and King, Saud University Residency Programs, Riyadh, Saudi Arabia

**Keywords:** Perceptions, Reflective logbooks, Residents, Dermatology, Saudi Arabia

## Abstract

**Background:**

Reflection is an essential feature of the learning process in all medical education and training fields. While writing the logbooks has been considered to improve learners’ reflection in several disciplines, it is unknown whether writing reflective logbooks improves the learning perception of dermatology residents. Therefore, this study was carried out to assess the role of the reflective logbook in improving the residents’ perception of reflective learning in the dermatology residency program.

**Methods:**

This cross-sectional study was conducted on 32 dermatology residents from year two (R2) to year four (R4) enrolled in different hospitals in Riyadh, Saudi Arabia. A baseline electronic survey was emailed to all residents before and after implementing the reflective logbook for six months. The survey included 28 close-ended questions assessing their point of view on a Likert scale, and responses varied from agreeing to disagreeing strongly. Frequencies and proportions were generated for the categorical variables such as sex and level of residency. The graphs were generated to assess the proportion of residents correctly responding to the knowledge questions. The data were analyzed using SPSS version 23.

**Results:**

Almost 80% of dermatology residents considered reflection as an important element of their learning, and 47% of the residents agreed that a reflective log book increases understanding in medical education. About 44% of the residents agreed that the reflective logbook assisted in their learning, and about 19% strongly agreed on the same. However, only 37.5% agreed that logbooks helped them acquire theoretical knowledge, and 9.4% and 18.8% agreed that they helped them acquire practical and research skills, respectively. Almost 68% of the residents had support in writing the logbook, and 34.4% agreed that logbooks helped them address their patient’s needs and enhance their diagnostic skills.

**Conclusion:**

The study findings revealed that dermatology residents perceived reflection and writing a reflective logbook as important and potentially beneficial. However, knowing the importance of reflection and what reflection means was not optimal. Not all dermatology residents were aware of the reflection and reflective logbooks as tools for reflective learning. Thereby warranting regular workshops or fostering continuous medical education on the importance of reflection.

## Introduction

Reflection, “ a serious thought or consideration,” is an important feature of the learning process in all medical education and training fields, such as undergraduate, postgraduate, and continuous medical education [1]. Reflection is a cognitive process in which new knowledge and experiences are integrated with previous acquaintances to understand oneself as well as surroundings so that one may meaningfully learn in the future based on the previous encounters and experiences [2]. By reflecting on the activities that a learner performs, one can learn from good points and maintain them in the future and also learn from mistakes and avoid repeating the same in the future [3, 4]. The importance of reflection cannot be overlooked in clinical training where clinicians learn by reflecting on their clinical encounters with patients or classroom exercises, or meetings with their mentors [1, 5]. Reflection occurs when learners in clinical practice are provided with an opportunity to think about and reflect on different aspects of learning such as training given to them, the research they undertake, policies or guidelines they follow in clinical practice and the education they receive during the training [6].

The benefits of reflection highlighted in the literature include the critical analysis and critical thinking of one’s attitudes, beliefs, and behaviors [7, 8]. In addition, reflection helps to learn complex subjects or areas and improves the level of comfort while exploring complex and difficult ideas [9]. Reflection may improve the clinicians’ performance and their critical thinking skills and help them learn more about how to communicate with patients, especially when a physician wants to break some bad news to the patients [10]. In addition, the reflection may help clinicians set their goals, meet patients’ needs, and contribute to a viable patient-clinician relationship [11]. In addition, this encounter with the patients also helps to enhance communication, interpersonal, diagnostic, and clinical skills during the training [12]. These skills are necessary to meet the needs of patients and have a good relationship with them. This can only be possible when learners reflect on what are the needs of patients, how they can improve their skills to meet those needs, what mistakes they should avoid or what good points they should consider enhancing their skills. In addition, the ongoing feedback from their mentors, for example, consultants, can help learners or trainers to reflect on their performance as they move on in the journey of learning [13].

The question arises of how self-reflection can be employed during the learning process. One of the answers could be to write down the points or take notes during their clinical rotation on which the learner can reflect later [14]. Writing is a process through which learners’ abilities to self-reflect on how and what they have learned to improve their active engagement with the process of learning [15]. For example, reflective logbooks and well-designed rubrics could be useful and effective for learners to write down their activities and reflect on their activities, followed by receiving continuous feedback and guidance from their mentors [10, 13, 16]. The literature reveals that logbooks are useful in continuous assessment and group learning [15, 17]. In addition, the logbooks encouraged interaction between the learners and mentors and were also a good tool for receiving and providing feedback [15, 17]. While the studies have assessed the role of logbooks in improving the reflection of students and learners in several disciplines, it is unknown whether writing reflective logbooks improve the learning perception of dermatology residents. Hence, this study was undertaken to assess the role of the reflective logbook in improving the residents’ perception of reflective learning in the dermatology residency program.

## Material and methods

### Study design, setting, and participants

This study was conducted on 32 dermatology residents from year two (R2) to year four (R4) practicing in various hospitals in Riyadh, Saudi Arabia. At the beginning of the academic year (October 2017), a reflective log book was emailed to 32 residents. So, inclusion criteria: All R2- R4 dermatology residents in Saudi Board of Dermatology residency program at Riyadh’s training hospitals. Exclusion criteria: R1 dermatology residents as they are practicing non dermatology clinical rotations in other specialties. Dermatology residents from other programs other than Riyadh’s training hospitals were excluded as the educators and trainers didn’t participate in the initiation of reflective logbooks project.

This reflective logbook was generated after reviewing the literature and scholarly publications to better understand what should be included in the reflective logbook. This was followed by training residents on how to fill the reflective log book with a special emphasis on the established outcomes and potential benefits of the implementation of the reflective logbook.

It was expected that each resident should fill the reflective logbook and write down their ideas about the patient’s encounter during two clinics per week. While filling the logbooks, the residents had to mention the important information such as medical records, number of patients, diagnosis, tasks assigned to them during clinical rotations, learning points, and self-learning. After residents wrote their logbooks, they got them reviewed by their faculty members in their respective institutions. The residents were given confidence and assurance about maintaining their privacy about their reflections, which allowed residents to not be fearful. Their respective mentors used to evaluate their reflections.

### Data collection

**Fig. 1:**
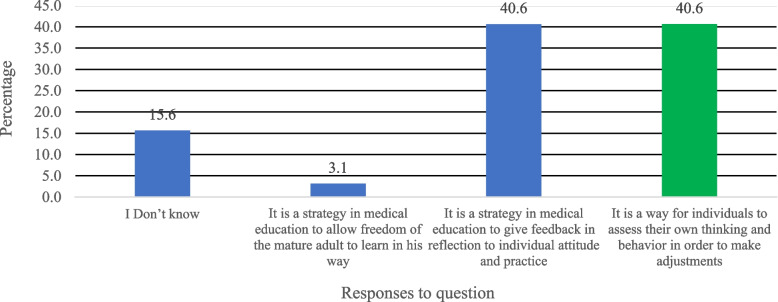
Knowledge assessment of dermatology residents: Response to Question on reflection in medical education

**Fig. 2:**
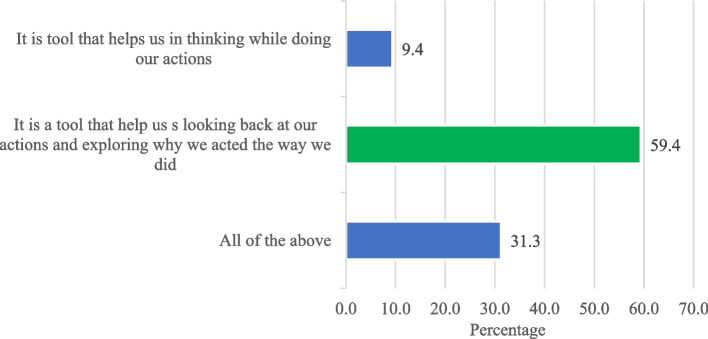
Knowledge assessment of dermatology residents: Statement expression more the reflective logbook

A baseline electronic survey was emailed to all residents involved in this study before implementing the reflective logbook. The same survey was repeated after six months of implementation of the reflective logbook. This survey had questions on the gender and level of residency. In addition, this survey also assessed the baseline knowledge of residents about the reflection. This was done through two questions in the survey. First: What is reflection in medical education? The second question: Which of the following statement express more the reflective logbook? With selection of one best answer of four options. (Figs. 1 and 2). Furthermore, this survey included 28 close-ended questions assessing their point of view on a Likert scale (single type questions), and responses varied from agreeing to strongly disagree. In addition, the residents were given a chance to mention additional comments or suggestions to improve the logbook and reflective meetings, if any, by responding to the open-ended questions. These questions and their responses on Likert scales examined residents’ perceptions towards reflection and reflective tools and their experience with the logbook and reflective meetings.

### Statistical methods

Since it was a descriptive study, the frequencies and proportions were generated for the categorical variables such as sex and level of residency. The graphs were generated to assess the proportion of residents correctly responding to two different knowledge questions on reflection. In addition, the frequencies and proportions were generated for all responses obtained on the Likert scale, and the frequencies and proportions were provided for the responses such as “agree,” “disagree,” “neutral,” “strongly agree,” and “strongly disagree.” The data were analyzed using SPSS software version 23.

#### Ethical consideration

The participants were informed about the research purpose and were required to sign an informed consent form. All data from participants was blinded and all data were identified using numbers. Approval was obtained from each participant before the contribution to the study. It was at the beginning of the study questioner. They were assured that their participation in this study is completely voluntary and that they can disenroll from the study at any time. If any participants had any questions, they would have contacted the principal investigator via email. Moreover, there was no incentive provided to the student for completing the survey. Also, intuition IRB approval was obtained by Chairman IRB Sub-Committee Health Sciences Colleges Research on Human Subjects King Saud University – College of Medicine. All methods were performed in accordance with relevant guidelines and regulations.

## Study results

### Sociodemographic characteristics of residents and their exposure to the reflective logbook

Table 1 provides the results on sociodemographic characteristics of residents and their exposure to the reflective logbook. The findings illustrated that an equal proportion of residents were of the same sex, i.e., 50% of residents were males, and 50% were females. With respect to the level of residency, more than a quarter of residents (28.1%) were from year 2, and a little over one-third of the residents were each from year three (37.5%) and year four (34.4%), respectively. While assessing their previous exposure to logbooks, more than half of the residents (53.1%) were exposed to the reflective logbook in the past. Further, 40.6% of the residents were exposed to a reflective logbook for three months, and 3.1% had been exposed to the logbook for at least four years. The results showed that almost 60% of the residents spent more than 60 min on logbooks per week, and one-third of the residents (34.4%) spent 15 to 60 min per week on logbooks, as illustrated in Table 1.

**Table 1:** Sociodemographic characteristics of residents and their exposure to the reflective logbook (*n* = 32)

Variable	n	%
Sex		
Female	16	50
Male	16	50
Level of residency		
R2	9	28.1
R3	12	37.5
R4	11	34.4
Previous exposure to a reflective logbook		
No	15	46.9
Yes	17	53.1
Years of exposure to the reflective logbook		
1 year	1	3.1
2 years	2	6.3
3 months	13	40.6
4 years and more	1	3.1
No	15	46.9
Time spent per week for a reflective logbook		
15—60 min	11	34.4
5—15 min	2	6.3
more than 60 min	19	59.4

### Knowledge testing of residents about reflective logbook

At the same time, we evaluated the knowledge of residents about the reflective logbook. The two graphs show the responses to two questions chosen to assess residents’ knowledge about the reflective logbook. The first question was” what is a reflection in medical education,” as shown in Fig. 1. About 41% of the residents responded correctly to the question by saying, “It is a way for individuals to assess their own thinking and behavior to make adjustments.” On the other hand, 15.6% did not know the answer to this question, and the remaining 45% marked the incorrect answer, as shown in Fig. 1.

Similarly, the second question of knowledge assessment asked, “Which of the following statements express more the reflective logbook?” as shown in Fig. 2. In response to this question, 59.4% responded that “it is a tool that helps us look back at our actions and explore why we acted the way we did,” and it was the correct answer to this question (Fig. 2). On the contrary, the remaining 41% marked incorrect answers to this question on knowledge assessment about reflective logbook, as shown in Fig. 2. The responses to these two questions indicate that not all dermatology residents were aware of the reflection and what reflection express.

Perceptions of residents about the importance of a reflective logbook in their residency program.

Table 2 shows the results on the perceptions of residents about the importance of a reflective logbook in their residency program. The responses were collected on a Likert scale ranging from agreeing to strongly disagree. While assessing their point of view on the importance of reflection in medical education, 50% of the residents agreed on this prompt, and one-third of the residents strongly agreed on the importance of reflection in medical education. This implies that dermatology residents consider reflection as an important element of their learning during training. Similarly, on assessing their views on whether a “Reflective log book increases understanding in medical education,” 47% of the residents agreed to this. However, almost 16% showed disagreement with this, and 9.4% showed strong disagreement with this. While assessing their reflective status on logbooks, it was found that only one-third of the residents showed agreement that logbooks made them more reflective than they are. More than a quarter of the residents strongly agree with these components, suggesting that the majority of the residents thought that the logbook improved their reflective skills. Similarly, near about half of the residents (46.9%) agreed logbook is an important part of the assessment in dermatology. However, about 19% did not agree with this, and about 10% showed strong disagreement.

**Table 2:** Perceptions of dermatology residents about the importance of a reflective logbook in their residency program (*n* = 32)

	Responses on the Likert scale									
	Agree		Disagree		Neutral		Strongly agree		Strongly disagree	
Items on the Likert scale	n	%	n	%	n	%	n	%	n	%
Reflection is important in medical education	16	50	3	9.4	1	3.1	10	31.3	2	6.3
Reflective log book increases understanding in medical education	15	47	5	16	4	12.5	5	15.6	3	9.4
Logbook makes me more reflective than I am	10	31	6	19	4	12.5	9	28.1	3	9.4
The logbook should be part of the assessment in dermatology	15	47	4	13	6	18.8	3	9.4	4	12.5
Logbook has assisted me in learning	14	44	2	6.3	5	15.6	6	18.8	5	15.6
Logbook has assisted me in acquiring theoretical knowledge	12	38	1	3.1	6	18.8	8	25	5	15.6
Logbook has assisted me in acquiring practical skills	3	9.4	10	31	10	31.3	4	12.5	5	15.6
Logbook has assisted me in identifying my learning needs	17	53	2	6.3	5	15.6	3	9.4	5	15.6
Learning by reflective logbook means time losing	3	9.4	10	31	9	28.1	8	25	2	6.3
Writing a log book helps me to get new ideas	11	34	5	16	6	18.8	7	21.9	3	9.4
Logbook has enhanced my research skills	6	19	8	25	8	25	3	9.4	7	21.9
I found it difficult to write a logbook	7	22	15	47	4	12.5	5	15.6	1	3.1
Writing a logbook decreases my stress	3	9.4	18	56	2	6.3	1	3.1	8	25
I need more support to do my logbook	17	53	2	6.3	7	21.9	5	15.6	1	3.1
Logbook can help me to address better patient's needs	11	34	5	16	5	15.6	4	12.5	7	21.9
Logbook enhances my diagnostic skills	11	34	3	9.4	8	25	3	9.4		21.9
The reflective logbook led to more clinical teaching by consultant	4	13	13	41	4	12.5	3	9.4	8	25
Prefer to have a log book next year	10	31	3	9.4	9	28.1	4	12.5	6	18.8
The logbook should be part of the residency program	12	38	3	9.4	8	25	4	12.5	5	15.6

In addition, the researchers assessed the resident’s viewpoints on the role of the logbook in their learning. About 44% of the residents agreed that the reflective logbook assisted in their learning, and about 19% strongly agreed on the same, as shown in Table 2. However, only one-third of the residents (37.5%) agreed that logbooks helped them acquire theoretical knowledge. Similarly, a small proportion of residents (9.4%) agreed that logbooks helped them to acquire any practical skills. This means that the majority of residents (31.3%) did not agree on the role of logbooks in improving their practical skills, and 15.6% showed strong disagreement on this. In contrast, more than half of the residents (53.1%) agreed that logbooks had assisted them in identifying their learning needs.

Almost one-third of the residents disagreed with the prompt, saying “learning by reflective logbook wastes their time”; however, 25% of the residents showed strong agreement with this. This suggests that almost a quarter of the residents considered this as losing time. Almost one-third of the residents agreed that writing a logbook is helpful to them to have new ideas, and 22% of the residents showed strong agreement on the same. Nevertheless, almost a quarter of the residents disagreed on the role of logbooks in enhancing their research skills and another quarter showed strong disagreement on the same. This indicates that logbooks may not be considered useful in improving their research skills. Similarly, the majority of the residents (46.9%) disagreed on the point that writing a logbook is difficult for them. However, a little less than a quarter of the residents (21.9%) agreed that it is difficult for them to write the logbook. Similarly, more than half of the residents (56.3%) disagreed on the role of logbook in reducing stress, and one quarter (25%) showed strong disagreement. This implies that writing a logbook may not be a fun activity for the residents; rather, it may be stressful.

While assessing if the residents need support in writing logbooks, it was found that 53.1% agreed that they need to have more support to do the reflective logbooks, and 15.6% strongly agreed on the same. Likewise, one-third of the residents (34.4%) agreed that logbooks helped them address their patient’s needs and enhanced their diagnostic skills. However, almost a quarter of the residents (21.9%) strongly disagree with this. Almost 41% of the residents disagreed with the idea that the reflective logbook leads to more clinical teaching by consultants, and 25% showed strong disagreement with the same idea. While assessing the preference of having a logbook next year, one-third of the residents (31.3%) agreed to have it next year. However, over a quarter of the residents (28.1%) were neutral, as demonstrated in Table 2. Similarly, 37.5% of the residents agreed that logbooks should be part of the residency program, and 12.5% strongly agreed (Table 2).

## Discussion

This cross-sectional study was conducted to assess the role of the reflective logbook in improving the year two to year four residents’ perception of reflective learning in the dermatology residency program. The findings revealed that not all residents were aware of the concept of reflection in medical education. Less than half of the residents marked correct answers when asked about the concept of medical education. While more than half of the residents marked correct answers when asked about what the reflective logbook expresses, it seems that a greater proportion of the residents were not able to answer this question. These findings reveal the need to increase the knowledge of dermatology residents about reflection and the importance of writing reflective logbooks in the residency program.

While assessing residents’ perceptions about the reflective logbook, residents acknowledged that reflection is important in medical education and reflective logbook increases their understanding of medical education. Further, majority of the residents agreed that the logbooks should be part of the assessment in dermatology as the logbook has assisted them in learning and in acquiring theoretical knowledge. Further, writing a logbook was found to be stressful for the majority of the residents. Ironically, almost half of the residents did not find it challenging to write a reflective logbook or learn by the log as a waste of time. This is in contrast to another study that aimed to assess students’ perceptions of the use of learning logs, where most students reported that learning logs are a time-consuming process [17]. Lastly, most of the residents in our study preferred to have a reflective logbook in the future years, and they thought that logbooks should be part of the residency program.

The importance of reflection is found in many disciplines that focus on the process of learning with better outcomes [18]. This process of reflection relates to the idea of looking back and assessing the experiences of the past to learn from what went well and avoid repeating mistakes [18]. However, few research studies on assessing the perceptions of students or residents on the importance of reflection and reflective logbooks have been conducted in different settings using the same Likert-type questions. Therefore, it was challenging for us to compare our study findings with other studies. For example, one study was conducted on third-year students in clinical emergency medicine in the MD program at Uppsala University to shed light on various aspects of reflection in medical education [19]. The importance of reflective writing in this study conducted at Uppsala University slightly differs from ours [19]. For example, 37% of the students agreed that the reflective logbook had increased their knowledge of reflection in medical education compared to 65% of the residents who agreed with the same response in our study [19]. Likewise, a little lower proportion of medical students at Uppsala University (42%) agrees that reflection is important in medical education than more than half of the residents have similar opinions in our study [19]. Similarly, almost one-third of the students (37.5%) believed that logbooks should become a permanent feature in their MD program, compared to 50% of the residents with similar opinions [19]. Less than one-third of students at Uppsala University spent about 15 to 60 min writing a logbook as opposed to 34.3% of residents in the current study [19]. Similarly, 90% of the students agreed that reflective logbooks had assisted them in learning practical skills, as opposed to 23% of the residents in the current study [19].

These differences in the proportions could be because of different levels of standard and training. Medical students may not have the same maturity, knowledge, and experience to understand the importance of reflection in medical education as dermatology residents at the next level of their training [20, 21]. Similarly, residents may need to take more time to write their reflections after having an encounter with their patients during clinical rotation. In contrast, medical students may not have enough clinical exposure to reflect on. Consequently, they may not take much time to write their reflections. Despite these differences in the findings of both studies, it seems that reflection and writing a reflective logbook are given importance in medical education and clinical practice.

## Strengths and limitations

This cross-sectional study was the first to explore residents’ perceptions of the importance of reflection and a reflective logbook. A validated and reliable tool was used to capture residents’ responses on the Likert scale. The findings of this study have important implications for residency programs in all specialties, and these findings can be used to integrate reflection and respective tools in training programs for residents. While the sample size appears to be smaller, we attempted to include dermatology residents from all clinical centers in Riyadh, Saudi Arabia. Usually, few residents opt for dermatology as compared to other specialties. Therefore, a sample size of 32 was found optimal in our study. In addition, the current study included dermatologists from all centers in Riyadh. Hence the study findings can at least be generalized to Riyadh and other similar cities in Saudi Arabia.

Despite these strengths, our study is not free from limitations, and findings should be interpreted considering these limitations. Typically to capture a construct or idea, there should be a variety of sub-questions under the main theme or question. Therefore, a possible threat to internal validity was single-type Likert-type questions, which may not truly reflect the underlying responses of residents. Another limitation of the current study is the study design, and because of the descriptive nature of the study design, the advanced analysis to identify the factors of inadequate knowledge about reflection, for example, could not be assessed. Further, the current study only evaluated residents’ perceptions and did not study the faculty’s perceptions on writing reflective logbooks and the importance of reflection. In the future, the faculty members should also be considered important stakeholders to contribute knowledge for similar studies.

### Conclusion and future implications

Clinicians and residents are expected to perform at a higher level in clinical life to meet the patient’s needs. To meet these needs, residents and trainees should have the means to reflect on their knowledge and practical skills. This study attempted to develop a reflective log and implemented the same on dermatology residents with the aim to collect data on residents’ perceptions of the reflective logbook utility and its use in the future for the dermatology residency program. We found that dermatology residents in all years perceived reflection and writing a reflective logbook as important and potentially beneficial. However, the knowledge of the importance of reflection and what reflection means was not optimal. Not all dermatology residents were aware of the reflection and reflective logbooks as tools for reflective learning. Thereby warranting mechanisms to enhance their knowledge through regular workshops or fostering continuous medical education on the importance of reflection.

While this study was not able to measure residents’ level of reflectivity with objective tools, the study’s findings provide a framework for the future to integrate reflective approaches in the training programs of clinicians. While the reflective logbook may be one tool for residents to reflect on their clinical skills and knowledge, consultants and faculty members in the residency programs can integrate the reflective logbook with group meetings to make the reflective program more meaningful and productive. Secondly, in the future, residency program management and leadership can consider accommodating diverse learning strategies and ways of reflection to provide a wide range of methods to express ideas and viewpoints of residents. Further, additional large studies are required to understand how reflection can improve learning and growth potential in residents by choosing more objective methods of assessment.

## Data Availability

The datasets used and/or analyzed during the current study available from the corresponding author on reasonable request.
